# Ethiopian Medicinal Plants Traditionally Used for the Treatment of Cancer; Part 3: Selective Cytotoxic Activity of 22 Plants against Human Cancer Cell Lines

**DOI:** 10.3390/molecules26123658

**Published:** 2021-06-15

**Authors:** Solomon Tesfaye, Hannah Braun, Kaleab Asres, Ephrem Engidawork, Anteneh Belete, Ilias Muhammad, Christian Schulze, Nadin Schultze, Sebastian Guenther, Patrick J. Bednarski

**Affiliations:** 1 School of Pharmacy, College of Health Sciences, Addis Ababa University, Churchill Street, Addis Ababa 1176, Ethiopia; soltesfa2010@gmail.com (S.T.); kaleab.asres@aau.edu.et (K.A.); ephrem.engidawork@aau.edu.et (E.E.); antbeletes@yahoo.com (A.B.); 2 Department of Pharmaceutical Biology, Institute of Pharmacy, University of Greifswald, 17489 Greifswald, Germany; hannah.braun1997@yahoo.com (H.B.); christian.schulze@uni-greifswald.de (C.S.); nadin.schultze@uni-greifswald.de (N.S.); 3 National Center for Natural Products Research, Research Institute of Pharmaceutical Sciences, School of Pharmacy, University of Mississippi, University, MS 38677, USA; milias@olemiss.edu; 4 Department of Medicinal Chemistry, Institute of Pharmacy, University of Greifswald, 17489 Greifswald, Germany

**Keywords:** Ethiopia, medicinal plants, cytotoxicity, extractions, cancer

## Abstract

Medicinal plants have been traditionally used to treat cancer in Ethiopia. However, very few studies have reported the in vitro anticancer activities of medicinal plants that are collected from different agro-ecological zones of Ethiopia. Hence, the main aim of this study was to screen the cytotoxic activities of 80% methanol extracts of 22 plants against human peripheral blood mononuclear cells (PBMCs), as well as human breast (MCF-7), lung (A427), bladder (RT-4), and cervical (SiSo) cancer cell lines. Active extracts were further screened against human large cell lung carcinoma (LCLC-103H), pancreatic cancer (DAN-G), ovarian cancer (A2780), and squamous cell carcinoma of the esophagus (KYSE-70) by using the crystal violet cell proliferation assay, while the vitality of the acute myeloid leukemia (HL-60) and histiocytic lymphoma (U-937) cell lines was monitored in the 3-(4,5-dimethylthiazol-2-yl)-2,5-diphenyl-2*H*-tetrazolium bromide (MTT) microtiter assay. *Euphorbia schimperiana*, *Acokanthera schimperi*, *Kniphofia foliosa*, and *Kalanchoe petitiana* exhibited potent antiproliferative activity against A427, RT-4, MCF-7, and SiSo cell lines, with IC_50_ values ranging from 1.85 ± 0.44 to 17.8 ± 2.31 µg/mL. Furthermore, these four extracts also showed potent antiproliferative activities against LCLC-103H, DAN-G, A2780, KYSE-70, HL-60, and U-937 cell lines, with IC_50_ values ranging from 0.086 to 27.06 ± 10.8 µg/mL. Hence, further studies focusing on bio-assay-guided isolation and structural elucidation of active cytotoxic compounds from these plants are warranted.

## 1. Introduction

Cancer is one of the leading causes of death worldwide. Mainly due to the rapid growth in the human population and increment in the prevalence of risk factors associated with economic transition, cancer incidence and death are rising in Africa [1]. Moreover, according to the GLOBOCAN 2018 report, cancer death is higher than cancer incidence in Africa, as compared to the rest of the world [2]. This might be due to the lack of an early cancer detection system, trained health care personal, treatment facilities, and access to anticancer drugs. Due to these challenges and higher treatment costs, patients in Ethiopia often choose to use traditional plant remedies as stand-alone or adjunct treatments.

Medicinal plants have been traditionally used in Ethiopia for the treatment of various diseases, including cancer [3,4,5]. However, the cytotoxic activities of plants that are traditionally used to treat cancer in Ethiopia have not been reported for samples collected in Ethiopia [6,7,8,9]. Only a few plants collected from Ethiopian geographic locations have been investigated so far for their antiproliferative/cytotoxic activities [10,11,12,13]. Therefore, in continuation with our previous studies [3,10] in which we reported ethnobotanical evidence of Ethiopian anticancer plants, as well as the cytotoxic activities of 21 plants against MV4-11 (human myeloid leukemia) cell line, we have further screened the cytotoxic activities of these and one additional plant (22 plants) against MCF-7, A427, RT-4, SiSo, LCLC-103H, DAN-G, A2780, KYSE-70, HL-60, and U-937 human cancer cell lines. The phytoconstituents, including anticancer compounds, previously reported from these active plants have also been discussed in this paper.

## 2. Results and Discussion

From 73 plants that were reported for their traditional anticancer use in our ethnobotanical survey [3], 22 were selected based on their ethnobotanical and chemotaxonomic data. The majority of selected plants belong to Lamiaceae (18.2%), Asteraceae (13.6%), and Euphorbiaceae (9.1%) families. The 80% methanolic extracts of different parts of these plants were tested for their cytotoxic activity against A427, MCF-7, RT-4, and SiSo human cancer cell lines and peripheral blood mononuclear cells (PBMCs) by using the crystal violet cell antiproliferation and MTT cell viability assays, respectively. The extracts were tested in primary screening at a concentration of 50 µg/mL. Four plant extracts—*A. schimperi*, *E. schimperiana*, *K. foliosa*, and *K. petitiana*—showed negative T/C_corr._ values, indicating relevant cytotoxic activity at 50 µg/mL (Table 1).

**Figure 1:**
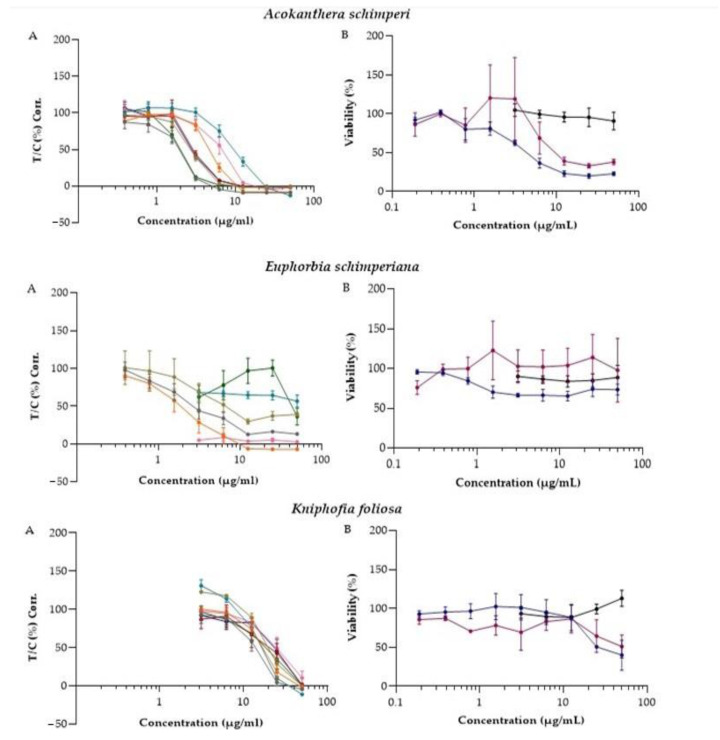

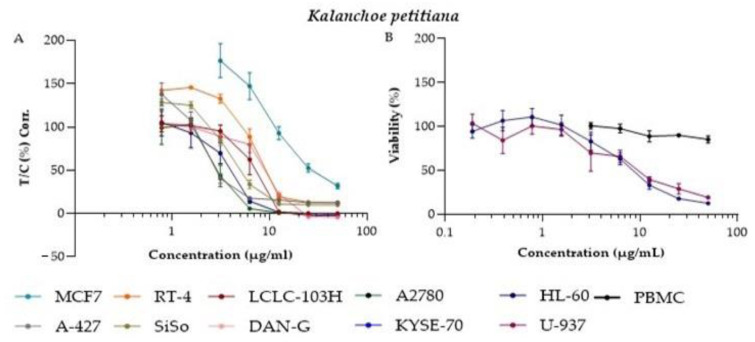
Antiproliferative and inhibitory effect on the viability of 4 extracts against 10 cancer cell lines and PBMCs: (**A**) concentration-response curve of *A. schimperi*, *E. schimperiana*, *K. foliosa,* and *K. petitiana* induced antiproliferative activity in MCF-7, A427, RT-4, SiSo, LCLC-103H, DAN-G, A2780, and KYSE-70 cell lines; (**B**) concentration-response curve of *A. schimperi*, *E. schimperiana*, *K. foliosa*, and *K. petitiana* induced inhibition of cell viability in HL-60 and U-937 cell lines as well as PBMC. Attachment and suspension cells were incubated with increasing concentrations of the extracts in culture medium for 96 h (crystal violet assay) and 24 h (MTT assay), respectively. Data are expressed as means ± SEM.

Based on the primary cytotoxic data, these four plants were selected for secondary screening at a range of concentrations against A427, MCF-7, RT-4, SiSo, and four additional adherents (LCLC-103H, DAN-G, KYSE-70, and A2780), and two suspensions (HL-60 and U-937) cell lines. PBMCs were included to compare the results obtained by the HL-60 and U-937 to primary noncancer cells. This should allow an initial assessment of the selectivity of the extracts. Accordingly, concentration-response curves of the extracts against 10 cell lines were generated and IC_50_ values were calculated (Figure 1, Table 2).

Selectivity is a desired property of active lead anticancer agents [14]. Different studies used PBMC as a model to check the cytotoxic effect of agents on normal human cells [15,16]. In the current study, tested extracts exhibited a much higher cytotoxic effect toward HL-60 and U-937 cell lines than PBMC. The cytotoxicity of all extracts against PBMC was greater than the highest concentration tested (IC_50_ ˃ 50 µg/mL).

*A. schimperi*: potent activity was observed when the extract of leaves of *A. schimperi* was tested on all ten cell lines with IC_50_ values ranging from 1.87 ± 0.4 to 10.31 ± 3.45 µg/mL. This plant is reported to contain acolongifloroside K as its major active principle, as well as cardiotonic glycosides ouabain and acovenoside A as minor constituents; in fact, it is used by Maasai people in east African as an arrow poison [17]. Young leaves of this plant are crushed and applied to the skin to treat cancer-like symptoms in the Ethiopian traditional medicinal system [3]. Furthermore, this plant has also been reported for its traditional use to treat wounds and hemorrhoids [18], hepatitis [19], and tonsillitis [20] in Ethiopia. Different crude extracts of *A. schimperi* have been reported for their antiviral [21], antibacterial [22], antiprotozoal [23], and in vitro cytotoxic [24] activities. Previous phytochemical studies have recorded the presence of flavonoids, terpenoids, tannins, cardiac glycosides, saponins, steroids, and carbohydrates [25].

*E. schimperiana*: the methanolic extract of *E. schimperiana* showed moderate cytotoxicity activity against the MCF-7 cell line, with an IC_50_ value of 25.2 µg/mL [26]. The methanol-water extracts of other species in the genus *Euphorbia* (*E. turcomanica*) also reduced the viability of HT-29 cells, with an IC_50_ value of 43 µg/mL [27]. Similarly, different solvent fractions of *E. umbellata* latex sap showed potent cytotoxic activity on hepatocellular carcinoma tumor cells (Hepa1c1c7), at IC_50_ values ranging from 2-12 µg/mL [28]. Euphol, a tetracyclic triterpene alcohol isolated from *E. tirucalli*, showed cytotoxic activity against a wide range of human cancer cell lines, including esophageal squamous cells (11.08 µM) and pancreatic carcinoma cells (6.84 µM) [29]. One triterpene 3β-cycloartenol and three phenolic compounds (chrysin, qurecetin-7-*O*-β-D glucoronside, and 3-methyl-qurecetin-7-*O*-β-D-glucoronside), isolated from the 80% methanol extract of *E. schimperiana*, showed antioxidant activities [30]. In the current study, *E. schimperiana* showed potent cytotoxic activity against A427, SiSo, and RT-4 cell lines at concentrations ranging from 1.85 ± 0.44 to 3.28 ± 1.2 µg/mL, and moderate cytotoxic activities against A2780 (26.54 ± 18.5 µg/mL).

*K. petitiana: K. petitiana*, known for its traditional use as a treatment of breast and skin cancer in Ethiopia [31], also showed promising cytotoxic activity against all cell lines used in our study, with IC_50_ values ranging from 2.09 ± 0.43 to 10.41 ± 5.59 µg/mL. Another plant from the same genus, *K. crenata*, has been reported to possess potent cytotoxic activity, with IC_50_ values of 2.33 and 28.96 µg/mL against mesothelioma (SPC212) and hepatocarcinoma (HepG2) cell lines, respectively [32]. The chloroform fractions of *K. gracilis*, on the other hand, showed only weak antiproliferative activity (IC_50_ = 136.85 ± 2.32 µg/mL) against human hepatocellular carcinoma (HepG2) [33]. Bryophyllin B, a bufadienolide isolated from *K. pinnata*, was reported for its potent cytotoxic activity against the human nasopharyngeal (KB) cell line, with the ED_50_ value of 80 ng/mL [34]. Furthermore, bufadienolide glycosides isolated from *K. tubiflora* were reported to displayed strong cytotoxic activity against A549, Cal-27 (oral adenosquamous carcinoma), A2058 (melanoma), and HL-60 (promyelocytic leukemia) cell lines [35].

*K. foliosa*: *K. foliosa* inhibited the proliferation of all cell lines, with IC_50_ values ranging from 14.54 ± 4.14 to 27.06 ± 10.8 µg/mL. Knipholone anthrone, a 4-phenylanthraquinone isolated from this plant, has been reported to possess cytotoxic activity against human acute monocytic (THP-1) and promonocytic (U-937) leukemic cell lines, with IC_50_ value of 0.9 ± 0.09 and 0.5 ± 0.05 µM, respectively [12].

## 3. Materials and Methods

### 3.1. Plant Material

**Figure 2:**
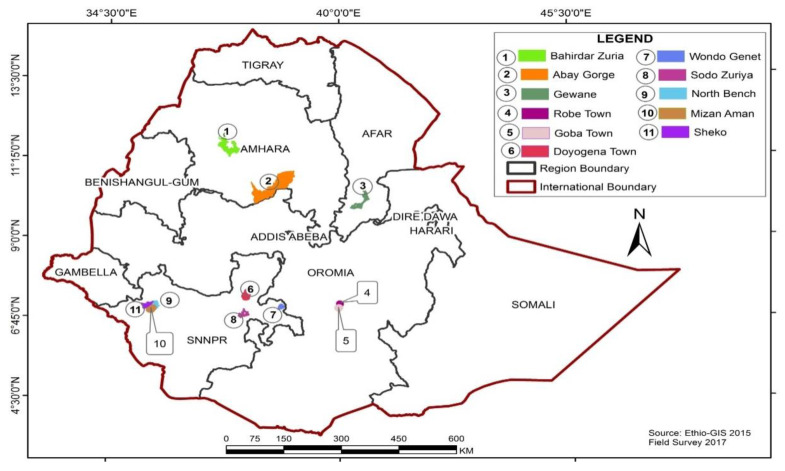
Map of Ethiopia showing the location of study districts.

Different parts of 22 plant species (Table 3) were collected from 9 districts, namely, Bale Robe, Bale Goba, Bahirdar Zuria, Abay Gorge, Gewane, Wondo Genet, Doyo Gena, North Bench, and Mizan Aman, of Ethiopia (Figure 2). These specimens were identified by a botanist (Mr. Melaku Wondafrash) and a voucher specimen of each plant was deposited at the National Herbarium, Addis Ababa University, Addis Ababa. All botanical names were transcribed according to the nomenclature system used by The Plant List (http://www.theplantlist.org, accessed on 15 April 2021). Each plant materials were shade dried and ground into powder.

### 3.2. Preparation of Crude Extract

The dried powder (200 g each) was macerated in 1 L (80% methanol) and shaken for 48 h. The macerated plant material was then filtered through Whatman No.1 filter paper by using a Buchner funnel. The crude methanol extracts were concentrated with a rotary evaporator (Büchi Rotavapor^®®^, R-200and R-210, Duisburg, Germany) with heating (Büchi heating bath^®®^, B-490 and B-491) at 37–40 °C, followed by freeze-drying (VaCo5, Zirbus Technology, Bad Grund, Germany) the aqueous concentrate. 

### 3.3. Cell Culture

The cancer cell lines in this study, MCF-7, A427, RT-4, SiSo, LCLC-103H, DAN-G, KYSE-70, A2780, HL-60, and U-937 were obtained from the German Collection of Microorganisms and Cell Culture (DMSZ Braunschweig, Germany) (Table 4). The A2780 cell line was provided by Dr. Julie A. Woods (Ninewells Hospital, University of Dundee, UK). These cell lines were routinely maintained in 75 cm^2^ culture flasks (Sarstedt, Nümbrecht, Germany), in a humid atmosphere of 5% CO_2_ at 37 °C [36]. Cells were grown in 90% RPMI-1640 media containing, 10% (*v*/*v*) heat-inactivated fetal bovine serum (Sigma-Aldrich, Munich, Germany) and supplemented with 30 mg/L penicillin and 40 mg/L streptomycin. Cells were incubated in a 5% CO_2_ humidified incubator (Heracell, Thermo Fisher Scientific, Waltham, MA, USA), at 37 °C, and passaged weekly.

### 3.4. Peripheral Blood Mononuclear Cell (PBMC) Isolation

Peripheral blood from healthy humans was provided by the blood bank of the University Medicine Greifswald. All donors signed informed consent forms that their blood could be used for research purposes, and all samples were randomized so as to conceal the identity of the donors. Blood samples were collected by venipuncture in tubes (BD Vacutainer^®®^) with 3.2% sodium citrate as an anticoagulant. Whole blood was 1:2 diluted with phosphate-buffered saline (PBS) (without Ca^2+^, Mg^2+^). Afterward, 10 mL Histopaque^®®^-1077 (Sigma-Aldrich, Munich, Germany) were overlaid with 10 mL of the diluted cell suspension and centrifuged without brake (400× *g*) for 30 min at room temperature. Subsequently, the PBMC layer was transferred to a new 50 mL centrifugal tube, washed with 10 mL PBS (without Ca^2+^, Mg^2+^), and centrifuged for 10 min at 250× *g*. The obtained cell pellet was then washed three times with PBS (5 mL) and centrifuged at 250× *g* for 10 min. After isolation, the PBMCs were cultured in RPMI 1640 (PAN-Biotech, Aidenbach, Germany), supplemented with 2 mM L-glutamine (Biochrom, Berlin, Germany) 1% *v*/*v* antibiotics (10,000 IU/mL penicillin; 10 mg/mL streptomycin, Sigma-Aldrich, Munich, Germany) and 10% *v*/*v* heat-inactivated fetal calf serum. The viability and number of cells were assessed by the trypan blue exclusion assay. Trypan blue was added in a 1:1 ratio to the cell suspension and counted with a Neubauer counting chamber. Cell viability was always >90%. Cells were adjusted to a density of 1 × 10^6^ cell/mL for the following MTT assay. The assay was performed as described, with extract concentrations ranging from 50 to 3.13 µg/mL.

### 3.5. Crystal Violet Cell Proliferation Assay

Crystal violet assay was used for both primary and secondary screening of extracts, as described previously [37]. This assay was used to test for the antiproliferative activity of the adherent cell lines. Briefly, each cancer cell line was seeded out in 96-well microtiter plates at a density of 1000 cells/well and incubated for 24 h to allow for attachment to the plate surface. The next day, the stock solution of each extract (20 µg/mL in dimethylsulfoxide) was serially diluted twofold to the desired concentration range, giving a series of five dilutions. Stock solutions and the dilutions were directly diluted 500-fold into the medium. From the working dilutions, 100 μL aliquots were added to each well. DMSO (0.1% (*v*/*v*) was used as a solvent. The plates were incubated for an additional 72 h at 37 °C. After 96 h, the culture medium was discarded and replaced with a 1% glutaraldehyde buffer saline for 20 min and then stored under Dulbecco’s buffer solution (pH 7.4) at 4 °C. On the day of staining, the buffer solution was removed, and the cells were stained with 0.02% crystal violet in deionized water (100 µL/well) for 30 min. Excess dye was discarded by washing the plates for 15 min in fresh water. The cell-bound dye was redissolved in 70% (*v*/*v*) ethanol/water and the optical density was measured at λ = 570 nm with a Spectramax 384 Plus plate reader (Molecular Devices, Sunnyvale, CA, USA) or a Sunrise plate reader (Tecan; Männedorf, Switzerland). The IC_50_ values were calculated by linear least-squares regression of the T/C corr values versus the logarithm of the added extract concentration and extrapolating to the T/C corr. values of 50% [38]. The corrected percent growth values (T/C) corr. (%) was calculated with the following equation:

(T/C) corr. (%) = (OD_T_ − OD_C__,__0_)/(OD_C_ − OD_C__,__0_) × 100 (1)

where OD_T_ is the mean optical density (OD) of the treated cells, OD_C_ the mean OD of the controls, and OD_C_,_0_ the mean OD of seeded cells at the time the drug was added.

### 3.6. MTT Cell Viability Assay

The 3-(4,5-dimethylthiazol-2-yl)-2,5-diphenyl-2*H*-tetrazolium bromide (MTT) assay was used in this experiment to determine the inhibition of the viability of suspension cells by the extracts [39]. Briefly, 20,000 cells were seeded out in 50 µL medium per well and immediately exposed to nine serial dilutions of extracts ranging from 50 to 0.19 µg/mL. After 24 h of incubation, 20 µL of freshly prepared solution of MTT in PBS (2.5 mg/mL) was added and the plates were returned to the incubator for an additional 4 h. Following the incubation, 100 µL of a 0.04 N HCl solution in isopropanol was added into each well, followed by sonication of the plates to dissolve the formed formazan crystals and the optical density was measured at λ = 570 nm with a Spectramax 384 Plus plate reader or a Sunrise plate reader (Tecan; Männedorf, Switzerland). Control experiments without cells were performed under the same assay conditions to rule out that the plant extracts themselves were reducing MTT to the blue formazan. No evidence for such a chemical reduction was observed in the OD readings, even at the highest extract concentrations.

### 3.7. Statistical Analysis

All tests were independently performed in triplicate. IC_50_ values were calculated with the software GraphPad Prism 7.0a by determining the inflection point of the simulated sigmoidal curves. The results are presented as means ± standard error of mean where appropriate.

## 4. Conclusions

The results of this study indicate that crude extracts of 4 out of the 22 plant species have good cytotoxic activity against human cancer cell lines, as depicted in Figure 1. Among these, four plants, *A. schimperi*, *E. schimperiana*, *K. petitiana* and *K. foliosa*, showed cytotoxic activity against all ten cell lines. Moreover, these extracts possessed selective cytotoxicity toward suspension cell lines (HL-60 and U-937) when compared to their effect on PBMC, consistent with their traditional use in anticancer therapies. In addition to these four plants, *C. abyssinica* and *G. involucrata* also showed selective cytotoxic/antiproliferative activities against some of the human cancer cell lines used in this study. These encouraging results have motivated us to begin isolating and identifying the active components of these four plant extracts, which may contain novel lead compounds for the treatment of cancer.

## Figures and Tables

**Table 1:** Cytotoxic activity T/C_corr_ (%) of extracts (50 µg/mL) after 96 h on MCF-7, A427, RT-4, and SiSo cell lines in primary screening. Testing was conducted with the crystal violet cell proliferation assay.

Extract	T/C_corr._ (%)			
	Cell Lines			
	A427	MCF-7	RT-4	SiSo
*A. schimperi*	−5.15	−14.96	−10.08	−1.37
*K. foliosa*	−5.76	−11.42	−7.19	−1.41
*K. petitiana*	−6.27	−7.64	−7.58	−2.13
*E. schimperiana*	−0.95	˃50	−10.41	30.77
*C. abyssinica*	29.29	˃50	−8.69	22.69
*G. involucrata*	49.65	˃50	29.17	˃50
*A. debrana*	26.76	˃50	˃50	˃50
*S. nilotica*	26.41	˃50	˃50	˃50
*C. simensis*	˃50	˃50	˃50	27.37
*T. schimperi*	˃50	˃50	˃50	32.79
*S. schimperiana*	46.62	˃50	˃50	˃50
*P. insipidum*	˃50	˃50	˃50	˃50
*A. caulirhiza*	˃50	˃50	˃50	˃50
*L. ocymifolia*	˃50	˃50	˃50	˃50
*D. barnimiana*	˃50	˃50	˃50	˃50
*R. nervosus*	˃50	˃50	˃50	˃50
*C. anisata*	˃50	˃50	˃50	˃50
*H. mannii*	˃50	˃50	˃50	˃50
*A. leucantha*	˃50	˃50	˃50	˃50
*V. auriculifera*	˃50	˃50	˃50	˃50
*C. brachycarpa*	˃50	˃50	˃50	˃50
*C. macrostachyus*	˃50	˃50	˃50	˃50

**Table 2:** IC_50_ values (µg/mL) for the activities of crude extracts against 10 human cancer MCF-7, A427, RT-4, SiSo, LCLC-103H, DAN-G, A2780, KYSE-70, HL-60, U-937 cell lines, and PBMC.

Cell Lines	Mean ± Standard Error of Mean (µg/mL)			
	*A. schimperi*	*E. schimperiana*	*K. petitiana*	*K. foliosa*
A427	2.17 ± 0.41	1.85 ± 0.44	2.09 ± 0.43	14.54 ± 4.14
MCF-7	10.31 ± 3.45	Nd	10.41 ± 5.59	14.89 ± 2.38
RT-4	5.18 ± 0.69	2.13 ± 3.78	6.83 ± 0.79	17.3 ± 5.44
SiSo	2.86 ± 0.29	3.28 ± 1.2	3.79 ± 0.49	17.8 ± 2.31
LCLC-103H	3.06 ± 0.3	0.086	7.33 ± 2.7	24.16 ± 0.4
DAN-G	5.23 ± 1.7	Nd	9.6 ± 1.6	27.06 ± 10.8
KYSE-70	2.87 ± 0.3	30.37	3.45 ± 1.6	22.03 ± 3.4
A2780	1.87 ± 0.4	26.54 ± 18.5	2.35 ± 0.9	16.77 ± 4.6
HL-60	4.08 ± 1.4	Nd	8.0 ± 1.7	24.2 ± 0.3
U-937	9.76 ± 6.8	47.68	8.58 ± 3.5	16.9
PBMC	˃50	˃50	˃50	˃50

Testing was conducted with the crystal violet assay except for the HL-60 and U937 cell lines, which were tested with the MTT assay. All values are averages with a standard error of mean of three independent experiments; Nd (not determined).

**Table 3:** List of 22 medicinal plants traditionally used to treat different human ailments in the study areas.

Voucher Number	Botanical Name (Family)	Vernacular Name	Districts	Growth Form	Parts Used
Bele-060	*Aloe debrana* (Xanthorrhoeaceae)	Gurtawaqota	DoyoGena	Shrub	Roots
Bel-002	*Hydrocotyle mannii* Hook.f (Apiaceae)	Ye’timedhanit	North Bench	Herb	Leaves
Bel-003	*Acokanthera schimperi* (A.DC.) Schweinf. (Apocynaceae)	Merenz	Bahir Dar Zuria	Shrub	Leaves
Bel-036	*Pentarrhinum insipidum* E.Mey. (Asclepiadaceae)	Barohula	Gewane	Shrub	Roots
Bel-020	*Kniphofia foliosa* Hochst. (Asphodelaceae)	Shushube	Bale Goba	Shrub	Roots
Bel-045	*Acmella caulirhiza* Delile (Asteraceae)	Kustasht	MizanAman	Shrub	Leaves
Bel-025	*Vernonia auriculifera* Hiern (Asteraceae)	Barawa	DoyoGena	Shrub	Leaves
Bel-021	*Cineraria abyssinica* Sch.Bip. ex A.Rich. (Asteraceae)	Esemefirh	Bale Robe	Herb	Leaves
Bel-039	*Cleome brachycarpa* (Forssk.) Vahl ex DC. (Capparidaceae)	Berbere	Gewane	Herb	Leaves
Bel-019	*Kalanchoe petitiana* A. Rich. (Crassulaceae)	Anchura	Bale Goba	Shrub	Leaves
Bel-032	*Euphorbia schimperiana* Scheele (Euphorbiaceae)	Gendalelata	DoyoGena	Shrub	Roots
Bel-035	*Croton macrostachyus* Hochst. ex Delile (Euphorbiaceae)	Besana	DoyoGena	Tree	Bark
Bel-043	*Ajuga leucantha* Lukhoba (Lamiaceae)	Tiksasht	North Bench	Herb	Leaves
Bel-024	*Leonotis ocymifolia* (Burm.f.) Iwarsson (Lamiaceae)	Armagusa	Bale Goba	Herb	Leaves
Bel-042	*Salvia nilotica* Juss. ex Jacq. (Lamiaceae)	Barnbanch	North Bench	Shrub	Whole plant
Bel-022	*Thymus schimperi* Ronniger (Lamiaceae)	Tosigne	Bale Goba	Herb	Leaves
Bel-051	*Sida schimperiana* Hochst. ex A. Rich. (Malvaceae)	Kotijebessa	Wondo Genet	Shrub	Roots and leaves
Bel-008	*Dorstenia barnimiana* Schweinf. (Moraceae)	Work Bemeda	Bahir Dar Zuria	Herb	Roots
Bel-018	*Rumex nervosus* Vahl (Polygonaceae)	Emboacho	Abay Gorge	Shrub	Roots
Bel-010	*Clematis simensis* Fresen. (Ranunculaceae)	YeazoHareg	Bahir Dar Zuria	Climber	Leaves
Bel-016	*Clausena anisata* (Willd.) Hook. f. ex Benth. (Rutaceae)	Limich	Abay Gorge	Shrub	Leaves
Bel-055	*Gnidia involucrata* Steud. ex A.Rich. (Thymelaeaceae)	Bito	Bahir Dar Zuria	Herb	Roots

**Table 4:** Cancer cell lines used.

Adherent Cell Lines	Corresponding Organ/Tissue
MCF-7	Breast Adenocarcinoma
A427	Lung Cancer
RT-4	Urinary bladder cancer
SiSo	Cervical Cancer
LCLC-103H	Large cell lung carcinoma
DAN-G	Pancreatic Cancer
A2780	Ovarian Cancer
KYSE-70	Squamous cell carcinoma of the esophagus
**Suspension Cell lines**	
HL-60	Acute myeloid leukemia
U-937	Histiocytic lymphoma

## Data Availability

The authors declare that all data supporting the finding of this study are included in this article.

## Sample Availability

Samples of the compounds are not available from the authors.
